# Supplementary material for: Unique Features and Collateral Immune Effects of mRNA-LNP COVID-19 Vaccines: Plausible Mechanisms of Adverse Events and Complications
Source: Pharmaceutics. 2025 Oct 13;17(10):1327. doi: 10.3390/pharmaceutics17101327 (PMC12567432; doi:10.3390/pharmaceutics17101327)
Supplement: Supplementary file 1 [file pharmaceutics-17-01327-s001.zip › pharmaceutics-3822729-supplementary.pdf]

Supplementary Table S1. Adverse events and complications of mRNA-LNP vaccines

Organ system	Adverse Event	References
Cardiovascular	Chest pain	[291–308]
	Arrhythmias	
	Myocarditis and pericarditis	
	Myocardial infarction	[309–311]
	Pulmonary embolism	[309,310]
	Thrombosis with thrombocytopenia	[310,312–320]
	Vasculitis	[321,322]
	Hypertension (severe)	[323,324]
Neurological	Guillain-Barré Syn (GBS)	[325–335]
	Bells' Palsy	[325,330,334–336]
	Encephalomyelitis	[325,330,334–339]
	Seizures	[325,330,334,335,340,341]
	Stroke	[309,310,315,316,319,320,325,330,342–344]
Immune	Inflammatory	[321,322,345]
	Autoimmune	[310,321,322,330,346–357]
	Allergic/ Pseudoallergic/ Anaphylactic	[322,358–362]
	Immunosuppression	[363–366]
Ocular		[322,367,368]
Death		[311,314,351,369]
Reproductive/Gynecological /Obstetric/Prenatal	Menstrual problems	[318,370–383]
Hematological	Coagulopathies	[317,318,321,351–353,383,384]
	Thrombotic events	[319,320,322,383]
	Hemophilia/hemolytic Anemia	[322]
	Lymphadenopathy	[385–387]
Dermatological	Erythema Multiforme	[385,388]
	Stevens-Johnson Syndr Epidermal Necrolysis	[358,359,361]
	Skin inflammation swelling	[389–391]
	Urticaria/flushing/rash	[310,322,354,358–361,392]
Rheumatological	Rheumatoid/ Polyarthritis	[322,357,393,394]
Respiratory	Acute Respiratory D Syndrome	[395]

Hepatobiliary	Hepatitis	[395–398]
Gastrointestinal<br>Hepatobiliary	Inflammatory	[395,399–401]
	Autoimmune	[395,396,400,401]
Renal/Urinary		[322,402–405]
Endocrine	Thyroiditis	[322,355,406,407]
	Diabetes	[356,408–411]
Oncological	Turbo cancer	[250–256,412]

References:

291. Gargano, J.W.; Wallace, M.; Hadler, S.C.; Langley, G.; Su, J.R.; Oster, M.E.; Broder, K.R.; Gee, J.; Weintraub, E.; Shimabukuro, T.; et al. Use of mRNA COVID-19 Vaccine After Reports of Myocarditis Among Vaccine Recipients: Update from the Advisory Committee on Immunization Practices—United States, June 2021. *Mmwr-Morbidity Mortal. Wkly. Rep.* **2021**, *70*, 977–982. https://doi.org/10.15585/mmwr.mm7027e2.
292. Hu, M.; Shoaibi, A.; Feng, Y.; Lloyd, P.C.; Wong, H.L.; Smith, E.R.; Amend, K.L.; Kline, A.; Beachler, D.C.; Gruber, J.F.; et al. Safety of Ancestral Monovalent BNT162b2, mRNA-1273, and NVX-CoV2373 COVID-19 Vaccines in US Children Aged 6 Months to 17 Years. *JAMA Netw. Open* **2024**, *7*, e248192–e248192. https://doi.org/10.1001/jamanetworkopen.2024.8192.
293. Eens, S., Van Hecke, M., Van den Bogaert, S., Favere, K., Cools, N., Fransen, E.; Roskams, T.; Heidbuchel, H.; Guns, P.J. A Murine Model of mRNA COVID-19 Vaccine-Induced Myocarditis: A Shot in the Dark? *JACC Basic Transl. Sci.* **2024**, *9*, 1026–28.
294. Zuin, M.; Zimelli, E.; Valle, C.D.; Cavedon, S.; Rigatelli, G.; Bilato, C. Diagnosis of Acute Myocarditis Following mRNA Vaccines against SARS-CoV-2: A Methodological Review. *Viruses* **2023**, *15*, 929. https://doi.org/10.3390/v15040929.
295. Zirkenbach, V.A.; Ignatz, R.M.; Öttl, R.; Cehreli, Z.; Stroikova, V.; Kaya, M.; Lehmann, L.H.; Preusch, M.R.; Frey, N.; Kaya, Z. Effect of SARS-CoV-2 mRNA-Vaccine on the Induction of Myocarditis in Different Murine Animal Models. *Int. J. Mol. Sci.* **2023**, *24*, 5011. https://doi.org/10.3390/ijms24055011.
296. Yeni, M. COVID-19 BNT162b2 mRNA vaccine induced myocarditis with left ventricular thrombus in a young male. *Acta Cardiol.* **2023**, *78*, 483–485. https://doi.org/10.1080/00015385.2023.2165271.
297. Yamada, T. Acute Myocarditis after the Third Dose of COVID-19 mRNA-1273 Vaccine. *J. Gen. Fam. Med.* **2023**, *24*, 188–89.
298. Weerts, V.; Lempereur, M.; Léonard, P.; Lancellotti, P. Facing COVID-19: myocarditis following vaccination with mRNA SARS-CoV-2. *Rev. Medicale Liege* **2023**, *78*, 141–146.
299. Wassif, M.; Lo, P.; Satouris, P.; Swan, L.; Tardo, D.; Kovacic, J.C.; Muller, D.; Muthiah, K.; Kotlyar, E.; Bart, N.K. Acute Myocarditis and Pericarditis after mRNA COVID-19 Vaccinations-a Single-Centre Retrospective Analysis. *Heart Lung Circ.* **2023**, *32*, 467–79.
300. Vila-Olives, R.; Uribarri, A.; Martínez-Martínez, M.; Argudo, E.; Bonilla, C.; Chiscano, L.; Herrador, L.; Gabaldón, A.; Buera, I.; Vidal, M.; et al. Fulminant myocarditis following SARS-CoV-2 mRNA vaccination rescued with venoarterial ECMO: A report of two cases. *Perfusion* **2023**, *39*, 655–659. https://doi.org/10.1177/02676591231170480.
301. Ulucay, A.S.; Singh, G.; Kanuri, S.H. Do COVID-19 viral infection and its mRNA vaccine carry an equivalent risk of myocarditis? Review of the current evidence, insights, and future directions. *Indian Hear. J.* **2023**, *75*, 217–223. https://doi.org/10.1016/j.ihj.2023.06.009.
302. Tome, J.; Cowan, L.T.; Fung, I.C.-H. A Pharmacoepidemiological Study of Myocarditis and Pericarditis Following the First Dose of mRNA COVID-19 Vaccine in Europe. *Microorganisms* **2023**, *11*, 1099. https://doi.org/10.3390/microorganisms11051099.

303. Straus, W.; Urdaneta, V.; Esposito, D.B.; A Mansi, J.; Rodriguez, C.S.; Burton, P.; Vega, J.M. Analysis of Myocarditis Among 252 Million mRNA-1273 Recipients Worldwide. *Clin. Infect. Dis.* **2023**, *76*, e544–e552. https://doi.org/10.1093/cid/ciac446.
304. Stowe, J.; Miller, E.; Andrews, N.; Whitaker, H.J. Risk of myocarditis and pericarditis after a COVID-19 mRNA vaccine booster and after COVID-19 in those with and without prior SARS-CoV-2 infection: A self-controlled case series analysis in England. *PLOS Med.* **2023**, *20*, e1004245. https://doi.org/10.1371/journal.pmed.1004245.
305. Sim, J.-Y.; Kim, S.-Y.; Kim, E.-K. The incidence and clinical characteristics of myocarditis and pericarditis following mRNA-based COVID-19 vaccination in Republic of Korea adolescents from July 2021 to September 2022. *Osong Public Heal. Res. Perspect.* **2023**, *14*, 76–88. https://doi.org/10.24171/j.phrp.2023.0032.
306. Shime, M.; Nozaki, Y.; Morita, A.; Ishiodori, T.; Murakami, T.; Yamasaki, H.; Yamamoto, M.; Takada, H. Life-Threatening Severe Acute Respiratory Syndrome Coronavirus-2 mRNA Vaccine-Associated Myocarditis after COVID-19 Myocarditis. *J. Paediatr. Child Health* **2023**, *59*, 1319.
307. Shenton, P.; Cheng, D.; Simm, P.; Jones, B.; Crawford, N. Myocarditis following COVID-19 mRNA vaccinations: Twin and sibling case series. *Vaccine X* **2023**, *14*, 100350. https://doi.org/10.1016/j.jvacx.2023.100350.
308. Altman, N.L.; Berning, A.A.; Mann, S.C.; Quaife, R.A.; Gill, E.A.; Auerbach, S.R.; Campbell, T.B.; Bristow, M.R. Vaccination-Associated Myocarditis and Myocardial Injury. *Circ. Res.* **2023**, *132*, 1338–57.
309. Jabagi, M.J.; Botton, J.; Bertrand, M.; Weill, A.; Farrington, P.; Zureik, M.; Dray-Spira, R. Myocardial Infarction, Stroke, and Pulmonary Embolism After BNT162b2 mRNA COVID-19 Vaccine in People Aged 75 Years or Older. *JAMA* **2022**, *327*, 80–82. https://doi.org/10.1001/jama.2021.21699.
310. Botton, J.; Jabagi, M.J.; Bertrand, M.; Baricault, B.; Drouin, J.; Le Vu, S.; Weill, A.; Farrington, P.; Zureik, M.; Dray-Spira, R. Risk for Myocardial Infarction, Stroke, and Pulmonary Embolism Following COVID-19 Vaccines in Adults Younger Than 75 Years in France. *Ann. Intern. Med.* **2022**, *175*, 1250–1257. https://doi.org/10.7326/m22-0988.
311. Liko, J.; Cieslak, P.R. Assessment of Risk for Sudden Cardiac Death Among Adolescents and Young Adults After Receipt of COVID-19 Vaccine—Oregon, June 2021–December 2022. *Mmwr—Morb. Mortal. Wkly. Rep.* **2024**, *73*, 317–320. https://doi.org/10.15585/mmwr.mm7314a5.
312. Parums, D.V. Editorial: SARS-CoV-2 mRNA Vaccines and the Possible Mechanism of Vaccine-Induced Immune Thrombotic Thrombocytopenia (VITT). *Med Sci. Monit.* **2021**, *27*, e932899–e2. https://doi.org/10.12659/msm.932899.
313. Rzymski, P.; Perek, B.; Flisiak, R. Thrombotic Thrombocytopenia after COVID-19 Vaccination: In Search of the Underlying Mechanism. *Vaccines* **2021**, *9*, 559. https://doi.org/10.3390/vaccines9060559.
314. Ostrowski, S.R.; Søgaard, O.S.; Tolstrup, M.; Stærke, N.B.; Lundgren, J.; Østergaard, L.; Hvas, A.-M. Inflammation and Platelet Activation After COVID-19 Vaccines—Possible Mechanisms Behind Vaccine-Induced Immune Thrombocytopenia and Thrombosis. *Front. Immunol.* **2021**, *12*, 779453. https://doi.org/10.3389/fimmu.2021.779453.
315. Yoshida, K.; Tanaka, K.; Suto, Y.; Fukuda, H. Repeated Cardioembolic Stroke after COVID-19 mRNA Vaccination: A Case Report. *J. Stroke Cerebrovasc. Dis.* **2022**, *31*, 106233–106233. https://doi.org/10.1016/j.jstrokecerebrovasdis.2021.106233.
316. Chui, C.S.L.; Fan, M.; Wan, E.Y.F.; Leung, M.T.Y.; Cheung, E.; Yan, V.K.C.; Gao, L.; Ghebremichael-Weldeselassie, Y.; Man, K.K.; Lau, K.K.; et al. Thromboembolic events and hemorrhagic stroke after mRNA (BNT162b2) and inactivated (CoronaVac) covid-19 vaccination: A self-controlled case series study. *eClinicalMedicine* **2022**, *50*, 101504. https://doi.org/10.1016/j.eclinm.2022.101504.
317. Gadi, S.R.V.; Brunker, P.A.R.; Al-Samkari, H.; Sykes, D.B.; Saff, R.R.; Lo, J.; Bendapudi, P.; Leaf, D.E.; Leaf, R.K. Severe autoimmune hemolytic anemia following receipt of SARS-CoV-2 mRNA vaccine. *Transfusion* **2021**, *61*, 3267–3271. https://doi.org/10.1111/trf.16672.
318. D'agostino, V.; Caranci, F.; Negro, A.; Piscitelli, V.; Tuccillo, B.; Fasano, F.; Sirabella, G.; Marano, I.; Granata, V.; Grassi, R.; et al. A Rare Case of Cerebral Venous Thrombosis and Disseminated Intravascular Coagulation Temporally Associated to the COVID-19 Vaccine Administration. *J. Pers. Med.* **2021**, *11*, 285. https://doi.org/10.3390/jpm11040285.

319. Ardalan, M.; Moslemi, M.; Pakmehr, A.; Vahed, S.Z.; Khalaji, A.; Moslemi, H.; Vahedi, A. TTP-like syndrome and its relationship with complement activation in critically ill patients with COVID-19: A cross-sectional study. *Heliyon* **2023**, *9*, e17370–e17370. https://doi.org/10.1016/j.heliyon.2023.e17370.
320. Ruggeri, T.; De Wit, Y.; Schärz, N.; van Mierlo, G.; Angelillo-Scherrer, A.; Brodard, J.; Schefold, J.C.; Hirzel, C.; Jongerius, I.; Zeerleder, S. Immunothrombosis and Complement Activation Contribute to Disease Severity and Adverse Outcome in COVID-19. *J. Innate Immun.* **2023**, *15*, 850–864. https://doi.org/10.1159/000533339.
321. Joob, B.; Wiwanitkit, V. COVID-19, Vaccination, Multisystem Inflammatory Syndrome, Aneurysm, Screening and Post Vaccination Death. *Int. J. Prev. Med.* **2023**, *14*, 74–74. https://doi.org/10.4103/ijpvm.ijpvm_192_21.
322. Afshar, Z.M.; Pirzaman, A.T.; Liang, J.J.; Sharma, A.; Pirzadeh, M.; Babazadeh, A.; Hashemi, E.; Deravi, N.; Abdi, S.; Allahgholipour, A.; et al. Do we miss rare adverse events induced by COVID-19 vaccination? *Front. Med.* **2022**, *9*, 933914. https://doi.org/10.3389/fmed.2022.933914.
323. Monadhel, H.; Abbas, A.; Mohammed, A. COVID-19 Vaccinations and Their Side Effects: A Scoping Systematic Review. *F1000Research* **2023**, *12*, 604.
324. Dzau, V.J.; Hodgkinson, C.P. RNA Therapeutics for the Cardiovascular System. *Circulation* **2024**, *149*, 707–716. https://doi.org/10.1161/circulationaha.123.067373.
325. Garg, R.K.; Paliwal, V.K. Spectrum of neurological complications following COVID-19 vaccination. *Neurol. Sci.* **2021**, *43*, 3–40. https://doi.org/10.1007/s10072-021-05662-9.
326. Ogata, S.; Ishii, Y.; Asano, K.; Kobayashi, E.; Kubota, S.; Takahashi, K.; Miyaji, Y.; Higashiyama, Y.; Joki, H.; Doi, H.; et al. Sensory Ataxic Guillain-Barré Syndrome with Dysgeusia after mRNA COVID-19 Vaccination. *Intern. Med.* **2022**, *61*, 1757–1760. https://doi.org/10.2169/internalmedicine.8967-21.
327. Hanson, K.E.; Goddard, K.; Lewis, N.; Fireman, B.; Myers, T.R.; Bakshi, N.; Weintraub, E.; Donahue, J.G.; Nelson, J.C.; Xu, S.; et al. Incidence of Guillain-Barré Syndrome After COVID-19 Vaccination in the Vaccine Safety Datalink. *JAMA Netw. Open* **2022**, *5*, e228879–e228879. https://doi.org/10.1001/jamanetworkopen.2022.8879.
328. Keh, R.Y.S.; Scanlon, S.; Datta-Nemdharry, P.; Donegan, K.; Cavanagh, S.; Foster, M.; Skelland, D.; Palmer, J.; Machado, P.M.; Keddie, S.; et al. COVID-19 vaccination and Guillain-Barré syndrome: analyses using the National Immunoglobulin Database. *Brain* **2023**, *146*, 739–748. https://doi.org/10.1093/brain/awac067.
329. Meo, S.A.; Shaikh, N.; Abukhalaf, F.A.; Meo, A.S. Exploring the adverse events of Oxford–AstraZeneca, Pfizer-BioNTech, Moderna, and Johnson and Johnson COVID-19 vaccination on Guillain–Barré Syndrome. *Sci. Rep.* **2024**, *14*, 1–14. https://doi.org/10.1038/s41598-024-66999-7.
330. de Sa, K.S.G.; Silva, J.; Bayarri-Olmos, R.; Brinda, R.; Constable, R.A.R.; Diaz, P.A.C.; Kwon, D.-I.; Rodrigues, G.; Wenxue, L.; Baker, C.; et al. A Causal Link between Autoantibodies and Neurological Symptoms in Long COVID. *medRxiv* **2024**. https://doi.org/10.1101/2024.06.18.24309100.
331. Ogunjimi, O.B.; Tsalamandris, G.; Paladini, A.; Varrassi, G.; Zis, P. Guillain-Barré Syndrome Induced by Vaccination Against COVID-19: A Systematic Review and Meta-Analysis. *Cureus* **2023**, *15*. https://doi.org/10.7759/cureus.37578.
332. Ha, J.; Park, S.; Kang, H.; Kyung, T.; Kim, N.; Kim, D.K.; Kim, H.; Bae, K.; Song, M.C.; Lee, K.J.; et al. Real-world data on the incidence and risk of Guillain–Barré syndrome following SARS-CoV-2 vaccination: a prospective surveillance study. *Sci. Rep.* **2023**, *13*, 1–9. https://doi.org/10.1038/s41598-023-30940-1.
333. Abara, W.E.; Gee, J.; Marquez, P.; Woo, J.; Myers, T.R.; DeSantis, A.; Baumblatt, J.A.G.; Woo, E.J.; Thompson, D.; Nair, N.; et al. Reports of Guillain-Barré Syndrome After COVID-19 Vaccination in the United States. *JAMA Netw. Open* **2023**, *6*, e2253845–e2253845. https://doi.org/10.1001/jamanetworkopen.2022.53845.
334. Hosseini, R.; Askari, N. A review of neurological side effects of COVID-19 vaccination. *Eur. J. Med Res.* **2023**, *28*, 1–8. https://doi.org/10.1186/s40001-023-00992-0.
335. Walker, J.L.; Schultze, A.; Tazare, J.; Tamborska, A.; Singh, B.; Donegan, K.; Stowe, J.; E Morton, C.; Hulme, W.J.; Curtis, H.J.; et al. Safety of COVID-19 vaccination and acute neurological events: A self-controlled case series in

England using the OpenSAFELY platform. *Vaccine* **2022**, *40*, 4479–4487. https://doi.org/10.1016/j.vaccine.2022.06.010.
336. Soeiro, T.; Salvo, F.; Pariente, A.; Grandvuillemin, A.; Jonville-Béra, A.-P.; Micallef, J. Type I interferons as the potential mechanism linking mRNA COVID-19 vaccines to Bell's palsy. *Therapies* **2021**, *76*, 365–367. https://doi.org/10.1016/j.therap.2021.03.005.
337. Morgan, H.J.; Clothier, H.J.; Kattan, G.S.; Boyd, J.H.; Buttery, J.P. Acute disseminated encephalomyelitis and transverse myelitis following COVID-19 vaccination—A self-controlled case series analysis. *Vaccine* **2024**, *42*, 2212–2219. https://doi.org/10.1016/j.vaccine.2024.01.099.
338. Poli, K.; Poli, S.; Ziemann, U. Multiple Autoimmune Syndromes Including Acute Disseminated Encephalomyelitis, Myasthenia Gravis, and Thyroiditis Following Messenger Ribonucleic Acid-Based COVID-19 Vaccination: A Case Report. *Front. Neurol.* **2022**, *13*, 913515. https://doi.org/10.3389/fneur.2022.913515.
339. Walter, A.; Kraemer, M. A neurologist's rhombencephalitis after comirnaty vaccination. A change of perspective. *Neurol. Res. Pr.* **2021**, *3*, 1–3. https://doi.org/10.1186/s42466-021-00156-7.
340. Núñez, I.; García-Grimshaw, M.; Valencia, C.Y.C.; Callejas, D.E.A.; Alfaro, M.L.M.; Saniger-Alba, M.d.M.; Gutiérrez-Romero, A.; Carrillo-Mezo, R.; Ceballos-Liceaga, S.E.; Baptista-Rosas, R.C.; et al. Seizures following COVID-19 vaccination in Mexico: A nationwide observational study. *Epilepsia* **2022**, *63*, e144–e149. https://doi.org/10.1111/epi.17390.
341. Doron, A.; Eviatar-Ribak, T.; Vituri, A.; Shahar, S.; Fahoum, F.; Goldstein, L. The COVID-19 pfizer BioNTech mRNA vaccine and the frequency of seizures. *Clin. Neurol. Neurosurg.* **2023**, *233*, 107952. https://doi.org/10.1016/j.clineuro.2023.107952.
342. Chemaitelly, H.; Akhtar, N.; Al Jerdi, S.; Kamran, S.; Joseph, S.; Morgan, D.; Uy, R.; Abid, F.B.; Al-Khal, A.; Bertollini, R.; et al. Association between COVID-19 vaccination and stroke: a nationwide case-control study in Qatar. *Int. J. Infect. Dis.* **2024**, *145*, 107095. https://doi.org/10.1016/j.ijid.2024.107095.
343. Lu, Y.; Matuska, K.; Nadimpalli, G.; Ma, Y.; Duma, N.; Zhang, H.T.; Chiang, Y.; Lyu, H.; Chillarige, Y.; Kelman, J.A.; et al. Stroke Risk After COVID-19 Bivalent Vaccination Among US Older Adults. *JAMA* **2024**, *331*, 938–950. https://doi.org/10.1001/jama.2024.1059.
344. Stefanou, M.I.; Palaiodimou, L.; de Sousa, D.A.; Theodorou, A.; Bakola, E.; Katsaros, D.E.; Halvatsiotis, P.; Tzavellas, E.; Naska, A.; Coutinho, J.M.; et al. Acute Arterial Ischemic Stroke Following COVID-19 Vaccination: A Systematic Review and Meta-Analysis. *Neurology* **2022**, *99*, e1465–e74.
345. Filippatos, F.; Tatsi, E.-B.; Michos, A. Immunology of Multisystem Inflammatory Syndrome after COVID-19 in Children: A Review of the Current Evidence. *Int. J. Mol. Sci.* **2023**, *24*, 5711. https://doi.org/10.3390/ijms24065711.
346. Efe, C.; Uzun, S.; Matter, M.S.; Beretta-Piccoli, B.T. Autoimmune-Like Hepatitis Related to SARS-CoV-2 Vaccination: Towards a Clearer Definition. *Liver Int.* **2025**, *45*, e16209.
347. Widhani, A.; Hasibuan, A.S.; Rismawati, R.; Maria, S.; Koesnoe, S.; Hermanadi, M.I.; Ophinni, Y.; Yamada, C.; Harimurti, K.; Sari, A.N.L.; et al. Efficacy, Immunogenicity, and Safety of COVID-19 Vaccines in Patients with Autoimmune Diseases: A Systematic Review and Meta-Analysis. *Vaccines* **2023**, *11*, 1456. https://doi.org/10.3390/vaccines11091456.
348. Hanberg, J.S.; Fu, X.; Wang, X.; Patel, N.J.; Kawano, Y.; Schiff, A.; Kowalski, E.N.; E Cook, C.; Vanni, K.M.M.; Guzzo, K.; et al. Effectiveness of a fourth dose of COVID-19 mRNA vaccine in patients with systemic autoimmune rheumatic diseases using disease-modifying antirheumatic drugs: an emulated target trial. *Lancet Rheumatol.* **2024**, *6*, e21–e30. https://doi.org/10.1016/s2665-9913(23)00272-2.
349. Kim, H.J.; Kim, M.H.; Park, S.J.; Choi, M.G.; Chun, E.M. Autoimmune Adverse Event Following COVID-19 Vaccination in Seoul, South Korea. *J. Allergy Clin. Immunol.* **2024**, *153*, 1711–20.
350. Wu, P.-C.; Huang, I.-H.; Wang, C.-Y.; Chi, C.-C. New Onset and Exacerbation of Autoimmune Bullous Dermatosis Following COVID-19 Vaccination: A Systematic Review. *Vaccines* **2024**, *12*, 465. https://doi.org/10.3390/vaccines12050465.

351. Fujisaki, Y.; Yasumi, M.; Shiraishi, K.; Kamijo, K.; Kamae, T.; Karasuno, T. Multiple microthromboses with autoimmune hemolytic anemia after BNT162b2 mRNA vaccination. *[Rinsho Ketsueki] Jpn. J. Clin. Hematol.* **2023**, *64*, 1421–1425. https://doi.org/10.11406/rinketsu.64.1421.
352. Nnawuba, K.C.; Boral, B.M.; Donnell, R.W. Probable warm autoimmune hemolytic anemia proceeding the administration of the Pfizer mRNA COVID-19 vaccine. *Immunohematology* **2022**, *38*, 106–107.
353. De Bruyne, S.; Van Landeghem, S.; Schauwvlieghe, A.; Noens, L. Life-Threatening Autoimmune Hemolytic Anemia Fol-lowing mRNA COVID-19 Vaccination: Don't Be Too Prudent with the Red Gold. *Clin. Chem. Lab. Med. (CCLM)* **2022**, *60*, e125–e28.
354. Atoui, A.; Jarrah, K.; Al Mahmasani, L.; Bou-Fakhredin, R.; Taher, A.T. Deep venous thrombosis and pulmonary embolism after COVID-19 mRNA vaccination. *Ann. Hematol.* **2022**, *101*, 1111–1113. https://doi.org/10.1007/s00277-021-04743-1.
355. Ciftel, S.; Tuzun, Z. Subacute Thyroiditis Following Sars-Cov-2 Vaccination: An Autoimmune/Inflammatory Syndrome Induced by Adjuvants (Asia Syndrome). *Acta Endocrinol. (Buchar.)* **2023**, *19*, 390–95.
356. Alsudais, A.S.; Alkanani, R.S.; Fathi, A.B.; Almuntashiri, S.S.; Jamjoom, J.N.; A Alzhrani, M.; Althubaiti, A.; Radi, S. Autoimmune diabetes mellitus after COVID-19 vaccination in adult population: a systematic review of case reports. *BMC Endocr. Disord.* **2023**, *23*, 1–9. https://doi.org/10.1186/s12902-023-01424-0.
357. Abe, N.; Bohgaki, M.; Kasahara, H. SARS-CoV-2 mRNA Vaccination-induced Autoimmune Polyarthritis-like Rheumatoid Arthritis. *Mayo Clin. Proc.* **2022**, *97*, 1574–1575. https://doi.org/10.1016/j.mayocp.2022.06.001.
358. Shah, M.M.; Layhadi, J.A.; Hourcade, D.E.; Fulton, W.T.; Tan, T.J.; Dunham, D.; Chang, I.; Vel, M.S.; Fernandes, A.; Lee, A.S.; et al. Elucidating allergic reaction mechanisms in response to SARS-CoV-2 mRNA vaccination in adults. *Allergy* **2024**, *79*, 2502–2523. https://doi.org/10.1111/all.16231.
359. Takata, H.; Shimizu, T.; Yamade, R.; Elsadek, N.E.; Emam, S.E.; Ando, H.; Ishima, Y.; Ishida, T. Anti-PEG IgM production induced by PEGylated liposomes as a function of administration route. *J. Control. Release* **2023**, *360*, 285–292. https://doi.org/10.1016/j.jconrel.2023.06.027.
360. Warren, C.M.; Snow, T.T.; Lee, A.S.; Shah, M.M.; Heider, A.; Blomkalns, A.; Betts, B.; Buzzanco, A.S.; Gonzalez, J.; Chinthrajah, R.S.; et al. Assessment of Allergic and Anaphylactic Reactions to mRNA COVID-19 Vaccines with Confirmatory Testing in a US Regional Health System. *JAMA Netw. Open* **2021**, *4*, e2125524–e2125524. https://doi.org/10.1001/jamanetworkopen.2021.25524.
361. Jaggers, J.; Wolfson, A.R. mRNA COVID-19 Vaccine Anaphylaxis: Epidemiology, Risk Factors, and Evaluation. *Curr. Allergy Asthma Rep.* **2023**, *23*, 195–200. https://doi.org/10.1007/s11882-023-01065-2.
362. Meroni, P.L.; Croci, S.; Lonati, P.A.; Pregnolato, F.; Spaggiari, L.; Besutti, G.; Bonacini, M.; Ferrigno, I.; Rossi, A.; Hetland, G.; et al. Complement activation predicts negative outcomes in COVID-19: The experience from Northen Italian patients. *Autoimmun. Rev.* **2022**, *22*, 103232. https://doi.org/10.1016/j.autrev.2022.103232.
363. Aochi, S.; Uehara, M.; Yamamoto, M. IgG4-related Disease Emerging after COVID-19 mRNA Vaccination. *Intern. Med.* **2023**, *62*, 1547–1551. https://doi.org/10.2169/internalmedicine.1125-22.
364. Hirano, H.; Asada, H. Exponential decline, ceiling effect, downregulation, and T-cell response in immunoglobulin G antibody levels after messenger RNA vaccine boosters: a case report. *J. Med Case Rep.* **2024**, *18*, 1–9. https://doi.org/10.1186/s13256-024-04889-2.
365. Amstutz, A.; Chammartin, F.; Audigé, A.; Eichenberger, A.L.; Braun, D.L.; Amico, P.; Stoeckle, M.P.; Hasse, B.; Papadimitriou-Olivgeris, M.; Manuel, O.; et al. Antibody and T-Cell Response to Bivalent Booster SARS-CoV-2 Vaccines in People With Compromised Immune Function: COVERALL-3 Study. *J. Infect. Dis.* **2024**, *230*, e847–e859. https://doi.org/10.1093/infdis/jiae291.
366. Qin, Z.; Bouteau, A.; Herbst, C.; Igyártó, B.Z. Pre-exposure to mRNA-LNP inhibits adaptive immune responses and alters innate immune fitness in an inheritable fashion. *PLOS Pathog.* **2022**, *18*, e1010830. https://doi.org/10.1371/journal.ppat.1010830.

367. Chau, C.Y.C.; Chow, L.L.W.; Sridhar, S.; Shih, K.C. Ophthalmological Considerations for COVID-19 Vaccination in Patients with Inflammatory Eye Diseases and Autoimmune Disorders. *Ophthalmol. Ther.* **2021**, *10*, 201–209. https://doi.org/10.1007/s40123-021-00338-1.
368. Han, J.Y.; Kim, S.; Han, J.; Kim, S.S.; Han, S.H.; Lee, S.W.; Kim, Y.J. Neuro-Ophthalmic Adverse Events of COVID-19 Infection and Vaccines: A Nationwide Cohort Study. *Investig. Ophthalmol. Vis. Sci.* **2023**, *64*, 37.
369. Tiede, A.; Sachs, U.J.; Czwalinna, A.; Werwitzke, S.; Bikker, R.; Krauss, J.K.; Donnerstag, F.G.; Weißenborn, K.; Höglinger, G.U.; Maasoumy, B.; et al. Prothrombotic immune thrombocytopenia after COVID-19 vaccination. *Blood* **2021**, *138*, 350–353. https://doi.org/10.1182/blood.2021011958.
370. Pietri, T.; Micallef, J.; Gervoise-Boyer, M.; Boyer, P. P-308 Spontaneous reports of menstrual cycle disorders after mRNA COVID-19 vaccine. *Hum. Reprod.* **2022**, *37* (Suppl. S1), deac107-294. https://doi.org/10.1093/humrep/deac107.294.
371. Chen, F.; Zhu, S.; Dai, Z.; Hao, L.; Luan, C.; Guo, Q.; Meng, C.; Zhang, Y. Effects of COVID-19 and mRNA vaccines on human fertility. *Hum. Reprod.* **2022**, *37*, 5–13. https://doi.org/10.1093/humrep/deab238.
372. Jorgensen, S.C.J.; Drover, S.S.M.; Fell, D.B.; Austin, P.C.; D'SOuza, R.; Guttmann, A.; A Buchan, S.; E Wilson, S.; Nasreen, S.; A Brown, K.; et al. Association between maternal mRNA covid-19 vaccination in early pregnancy and major congenital anomalies in offspring: population based cohort study with sibling matched analysis. *BMJ Med.* **2024**, *3*, e000743. https://doi.org/10.1136/bmjmed-2023-000743.
373. Fell, D.B.; Dhinsa, T.; Alton, G.D.; Török, E.; Dimanlig-Cruz, S.; Regan, A.K.; Sprague, A.E.; Buchan, S.A.; Kwong, J.C.; Wilson, S.E.; et al. Association of COVID-19 Vaccination in Pregnancy With Adverse Peripartum Outcomes. *JAMA* **2022**, *327*, 1478–1487. https://doi.org/10.1001/jama.2022.4255.
374. Ruderman, R.S.; Mormol, J.; Trawick, E.; Perry, M.F.; Allen, E.C.; Millan, D.; Miller, E.S. Association of COVID-19 Vaccination During Early Pregnancy With Risk of Congenital Fetal Anomalies. *JAMA Pediatr.* **2022**, *176*, 717–719. https://doi.org/10.1001/jamapediatrics.2022.0164.
375. Shi, W.; Wang, M.; Xue, X.; Li, N.; Chen, L.; Shi, J. Association Between Time Interval from COVID-19 Vaccination to In Vitro Fertilization and Pregnancy Rate After Fresh Embryo Transfer. *JAMA Netw. Open* **2022**, *5*, e2236609–e2236609. https://doi.org/10.1001/jamanetworkopen.2022.36609.
376. Zhao, Y.; Zhao, Y.; Zhang, Y.; Li, M.; Su, X.; Zhou, Y.; Zhang, Z.; Jin, L. Association of COVID-19 vaccination before conception with maternal thyroid function during early pregnancy: A single-center study in China. *J. Med Virol.* **2023**, *95*, e28245. https://doi.org/10.1002/jmv.28245.
377. Wong, J.Y.; Elwood, C.; Money, D.M.; Dunne, C. Myths Versus Facts: COVID-19 Vaccine Effects on Pregnancy, Fertility, and Menstruation. *Manag. Menopause New Guidel.* **2022**, *64*, 354–358.
378. Barros, F.C.; Gunier, R.B.; Rego, A.; Sentilhes, L.; Rauch, S.; Gandino, S.; Teji, J.S.; Thornton, J.G.; Kachikis, A.B.; Nieto, R.; et al. Maternal vaccination against COVID-19 and neonatal outcomes during Omicron: INTERCOVID-2022 study. *Am. J. Obstet. Gynecol.* **2024**, *231*, 460.e1–460.e17. https://doi.org/10.1016/j.ajog.2024.02.008.
379. Peretz-Machluf, R.; Gilboa, M.; Bookstein-Peretz, S.; Segal, O.; Regev, N.; Meyer, R.; Regev-Yochay, G.; Yinon, Y.; Toussia-Cohen, S. Obstetric and Early Neonatal Outcomes Following Second and Third COVID-19 Vaccination in Pregnancy. *Isr. Med. Assoc. J. IMAJ* **2024**, *26*, 12–17.
380. Santimano, A.J.; Al-Zoubi, R.M.; Al-Qudimat, A.R.; Al Darwish, M.B.; Ojha, L.K.; Rejeb, M.A.; Hamad, Y.; Elrashid, M.A.; Ruxshan, N.M.; El Omri, A.; et al. Efficacy and Clinical Outcomes of mRNA COVID-19 Vaccine in Pregnancy: A Systematic Review and Meta-Analysis. *Intervirology* **2024**, *67*, 40–54. https://doi.org/10.1159/000538135.
381. Moro, P.L.; Carlock, G.; Fifadara, N.; Habenicht, T.; Zhang, B.; Strid, P.; Marquez, P. Safety Monitoring of Bivalent mRNA COVID-19 Vaccine among Pregnant Persons in the Vaccine Adverse Event Reporting System—United States, September 1, 2022–March 31, 2023. *Vaccine* **2024**, *42*, 2380–84.

382. Choi, Y.J.; Jung, J.; Kang, M.; Choi, M.J.; Choi, W.S.; Bin Seo, Y.; Hyun, H.-J.; Yoon, Y.; Choe, Y.J.; Cho, G.J.; et al. The risk of pregnancy-related adverse outcomes after COVID-19 vaccination: Propensity score-matched analysis with influenza vaccination. *Vaccine* **2025**, *44*, 126506. https://doi.org/10.1016/j.vaccine.2024.126506.
383. Ghanbari, E.P.; Jakobs, K.; Puccini, M.; Reinshagen, L.; Friebel, J.; Haghikia, A.; Kränkel, N.; Landmesser, U.; Rauch-Kröhnert, U. The Role of NETosis and Complement Activation in COVID-19-Associated Coagulopathies. *Biomedicines* **2023**, *11*, 1371. https://doi.org/10.3390/biomedicines11051371.
384. Brito, S.; Ferreira, N.; Mateus, S.; Bernardo, M.; Pinto, B.; Lourenço, A.; Grenho, F. A Case of Autoimmune Hemolytic Anemia Following COVID-19 Messenger Ribonucleic Acid Vaccination. *Cureus* **2021**, *13*. https://doi.org/10.7759/cureus.15035.
385. Ng, C.; Tay, E.; D'Souza, A. Localised swelling at sites of dermal filler injections following administration of COVID-19 vaccines: a systematic review. *Singap. Med J.* **2024**, *65*, 665–668. https://doi.org/10.4103/singaporemedj.smj-2021-157.
386. Jue, M.-S.; Joh, H.C.; Kim, S.H.; Ko, J.Y. Stevens–Johnson Syndrome/Toxic Epidermal Necrolysis Overlap After the Third Dose of BNT162b2 mRNA COVID-19 Vaccination and Literature Review. *Dermatitis®* **2023**, *34*, 158–159. https://doi.org/10.1089/derm.2022.29003.msj.
387. Franzblau, L.E.; Mauskar, M.; Wysocki, C.A. Macrophage Activation Syndrome Complicated by Toxic Epidermal Necrolysis Following SARS-CoV-2 mRNA Vaccination. *J. Clin. Immunol.* **2023**, *43*, 521–524. https://doi.org/10.1007/s10875-022-01408-0.
388. Beamish, I.V.; Bogoch, I.I.; Carr, D. Delayed Inflammatory Reaction to Dermal Fillers after COVID-19 Vaccination: A Case Report. *Can. J. Emerg. Med.* **2022**, *24*, 444–46.
389. Yoshimura, Y.; Sasaki, H.; Miyata, N.; Miyazaki, K.; Okudela, K.; Tateishi, Y.; Hayashi, H.; Kawana-Tachikawa, A.; Iwashita, H.; Maeda, K.; et al. An autopsy case of COVID-19-like acute respiratory distress syndrome after mRNA-1273 SARS-CoV-2 vaccination. *Int. J. Infect. Dis.* **2022**, *121*, 98–101. https://doi.org/10.1016/j.ijid.2022.04.057.
390. Lopatynsky-Reyes, E.Z.; Acosta-Lazo, H.; Ulloa-Gutierrez, R.; Ávila-Aguero, M.L.; Chacon-Cruz, E. BCG Scar Local Skin Inflammation as a Novel Reaction Following mRNA COVID-19 Vaccines in Two International Healthcare Workers. *Cureus* **2021**, *13*. https://doi.org/10.7759/cureus.14453.
391. Ben-Fredj, N.; Chahed, F.; Ben-Fadhel, N.; Mansour, K.; Ben-Romdhane, H.; El Mabrouk, R.S.; Chadli, Z.; Ghedira, D.; Belhadjali, H.; Chaabane, A.; et al. Case series of chronic spontaneous urticaria following COVID-19 vaccines: an unusual skin manifestation. *Eur. J. Clin. Pharmacol.* **2022**, *78*, 1959–1964. https://doi.org/10.1007/s00228-022-03399-8.
392. Grieco, T.; Ambrosio, L.; Trovato, F.; Vitiello, M.; Demofonte, I.; Fanto, M.; Paolino, G.; Pellacani, G. Effects of Vaccination against COVID-19 in Chronic Spontaneous and Inducible Urticaria (CSU/CIU) Patients: A Monocentric Study. *J. Clin. Med.* **2022**, *11*, 1822. https://doi.org/10.3390/jcm11071822.
393. Iwamura, N.; Eguchi, K.; Takatani, A.; Tsutsumi, K.; Koga, T.; Araki, T.; Aramaki, T.; Terada, K.; Ueki, Y. A Case Series of Rheumatoid Arthritis Flare Including Extra-Articular Manifestations Following Sars-Cov-2 mRNA Vaccination: A Compre-hensive Cytokine Assay. *Cureus* **2024**, *16*, e58740.
394. Fong, W.; Woon, T.H.; Chew, L.-C.; Low, A.; Law, A.; Poh, Y.J.; Yeo, S.I.; Leung, Y.Y.; Ma, M.; Santosa, A.; et al. Prevalence and factors associated with flares following COVID-19 mRNA vaccination in patients with rheumatoid arthritis, psoriatic arthritis and spondyloarthritis: a national cohort study. *Hortic. Bras.* **2023**, *63*, 1–9. https://doi.org/10.1186/s42358-023-00316-0.
395. Lee, S.; Lee, K.; Park, J.; Jeong, Y.D.; Jo, H.; Kim, S.; Woo, S.; Son, Y.; Kim, H.J.; Lee, K.; et al. Global burden of vaccine-associated hepatobiliary and gastrointestinal adverse drug reactions, 1967–2023: A comprehensive analysis of the international pharmacovigilance database. *J. Med Virol.* **2024**, *96*, e29792. https://doi.org/10.1002/jmv.29792.
396. Kim, J.H.; Oh, E.H.; Han, D.S. Polyposis of gastrointestinal tract after COVID-19 mRNA vaccination: a report of two cases. *Clin. Endosc.* **2024**, *57*, 402–406. https://doi.org/10.5946/ce.2023.268.

397. Mujukian, A.; Kumar, R.; Li, D.; Debbas, P.; Botwin, G.J.; Cheng, S.; Ebinger, J.; Braun, J.; McGovern, D.; Melmed, G.Y.; et al. Postvaccination Symptoms After SARS-CoV-2 mRNA Vaccination Among Patients With Inflammatory Bowel Disease: A Prospective, Comparative Study. *Inflamm. Bowel Dis.* **2024**, *30*, 602–616. https://doi.org/10.1093/ibd/izad114.
398. Kaplan, B.; Coscia, G.; Fishbein, J.S.; Innamorato, A.; Ali, A.; Farzan, S. Gastrointestinal reflux contributes to laryngopharyngeal symptoms that mimic anaphylaxis: COVID-19 vaccination experience. *J. Allergy Clin. Immunol. Glob.* **2024**, *3*, 100176. https://doi.org/10.1016/j.jacig.2023.100176.
399. Murray, S.M.; Pose, E.; Wittner, M.; Londoño, M.-C.; Schaub, G.; Cook, J.; Dimitriadis, S.; Meacham, G.; Irwin, S.; Lim, Z.; et al. Immune responses and clinical outcomes after COVID-19 vaccination in patients with liver disease and liver transplant recipients. *J. Hepatol.* **2024**, *80*, 109–123. https://doi.org/10.1016/j.jhep.2023.10.009.
400. Taida, T.; Kato, J.; Ishikawa, K.; Akizue, N.; Ohta, Y.; Okimoto, K.; Saito, K.; Matsusaka, K.; Matsumura, T.; Kato, N. Severe ulcerative colitis induced by COVID-19 vaccination. *Clin. J. Gastroenterol.* **2024**, *17*, 447–450. https://doi.org/10.1007/s12328-024-01926-x.
401. Cannatelli, R.; Ferretti, F.; Carmagnola, S.; Bergna, I.M.B.; Monico, M.C.; Maconi, G.; Ardizzone, S. Risk of adverse events and reported clinical relapse after COVID-19 vaccination in patients with IBD. *Gut* **2022**, *71*, 1926–1928. https://doi.org/10.1136/gutjnl-2021-326237.
402. Eguchi, G.; Murakoshi, M.; Miyaoka, F.; Shimbo, A.; Irabu, H.; Kanamori, T.; Udagawa, T.; Morio, T.; Shimizu, M. Acute tubulointerstitial nephritis following coronavirus disease 2019 mRNA vaccination: a pediatric case report. *CEN Case Rep.* **2024**, *14*, 261–265. https://doi.org/10.1007/s13730-024-00945-4.
403. Abramson, M.; Yu, S.M.-W.; Campbell, K.N.; Chung, M.; Salem, F. IgA Nephropathy After SARS-CoV-2 Vaccination. *Kidney Med.* **2021**, *3*, 860–863. https://doi.org/10.1016/j.xkme.2021.05.002.
404. Shim, S.R.; Kim, K.T.; Park, E.; Pyun, J.H.; Kim, J.H.; Chung, B.I. Urological Complications after COVID 19 Vaccine Ac-cording to Age, Sex and Manufacturer. *World J. Urol.* **2023**, *41*, 2255–63.
405. Imhof, C.; Messchendorp, A.L.; Bungener, L.B.; Hepkema, B.G.; Kho, M.M.L.; Reinders, M.E.J.; Bemelman, F.J.; Hilbrands, L.B.; Gansevoort, R.T.; Sanders, J.S.F.; et al. The effect of COVID-19 vaccination on kidney function and HLA antibody formation in patients with end-stage kidney disease and on kidney replacement treatment. *Clin. Kidney J.* **2024**, *17*, sfae122. https://doi.org/10.1093/ckj/sfae122.
406. Kaur, U.; Reddy, N.T.S.; Reddy, J.; Krishna, D.V.V.; Dehade, A.; Agrawal, N.K. Patterns and outcomes of late onset thyroid disturbances after COVID-19 vaccination: A report of 75 cases. *Trop. Med. Int. Health* **2024**, *29*, 63–71. https://doi.org/10.1111/tmi.13947.
407. Jafarzadeh, A.; Nemati, M.; Jafarzadeh, S.; Nozari, P.; Mortazavi, S.M.J. Thyroid dysfunction following vaccination with COVID-19 vaccines: a basic review of the preliminary evidence. *J. Endocrinol. Investig.* **2022**, *45*, 1835–1863. https://doi.org/10.1007/s40618-022-01786-7.
408. Aburisheh, K.H.; Enabi, H.M.; Alodah, N.A.; Alotary, B.H.; Algheryafi, L.A.; Almairi, A.M.; Aldhewaila, A.A. New Onset of Type 1 Diabetes Mellitus Post-COVID-19 Vaccine. *J. Med. Cases* **2024**, *15*, 367–370. https://doi.org/10.14740/jmc4307.
409. Mungmunpuntipantip, R.; Wiwanitkit, V. Type 1 diabetes mellitus following SARS-CoV-2 mRNA vaccination. *Endocrine* **2022**, *78*, 641–641. https://doi.org/10.1007/s12020-022-03169-7.
410. Aydoğan, B.İ.; Ünlütürk, U.; Cesur, M. Type 1 diabetes mellitus following SARS-CoV-2 mRNA vaccination. *Endocrine* **2022**, *78*, 42–46. https://doi.org/10.1007/s12020-022-03130-8.
411. Xiong, X.; Lui, D.T.W.; Chung, M.S.H.; Au, I.C.H.; Lai, F.T.T.; Wan, E.Y.F.; Chui, C.S.L.; Li, X.; Cheng, F.W.T.; Cheung, C.-L.; et al. Incidence of diabetes following COVID-19 vaccination and SARS-CoV-2 infection in Hong Kong: A population-based cohort study. *PLOS Med.* **2023**, *20*, e1004274. https://doi.org/10.1371/journal.pmed.1004274.
412. Wang, Y.; Chen, D.; Pan, Y.; Li, H.; Zhao, W.; Lu, T.; Kong, W.; Ding, M.; Wang, X.; Zhang, G. Serological response and immune-related adverse events following COVID-19 vaccination in cancer patients treated with immune

checkpoint inhibitors: A systematic review and meta-analysis. *Rev. Med Virol.* **2024**, *34*. https://doi.org/10.1002/rmv.2495.

Disclaimer/Publisher's Note: The statements, opinions and data contained in all publications are solely those of the individual author(s) and contributor(s) and not of MDPI and/or the editor(s). MDPI and/or the editor(s) disclaim responsibility for any injury to people or property resulting from any ideas, methods, instructions or products referred to in the content.
